# Analysis of Top 100 Articles Cited in Top Pain Journals: A Comprehensive Two Decade Bibliometric Analysis

**DOI:** 10.1007/s11916-024-01273-z

**Published:** 2024-06-14

**Authors:** Kunal Vij, John M. Thomas, Leo Wan, Ajay N. Chatim, George Thomas, Alan D. Kaye

**Affiliations:** 1 West Virginia School of Osteopathic Medicine, Lewisburg, WV 24901 USA; 2 New York Institute of Technology College of Osteopathic Medicine, Old Westbury, NY. 11568 USA; 3 Department of Orthopaedic Surgery, University of Minnesota, Minneapolis, MN 55455 USA; 4 Departments of Anesthesiology and Pharmacology, Toxicology and Neurosciences, Louisiana State Health Sciences Center at Shreveport, Shreveport, LA 71103 USA

**Keywords:** Pain, Manuscript, Pain physician journal, Pain medicine, Regional anesthesia and pain medicine, Journal of pain

## Abstract

**Purpose of Review:**

A bibliometric analysis was performed to analyze and compare the top 100 articles from the most well-known five pain journals: *Pain*, *Pain Physician*, *Pain Medicine*, *Regional Anesthesia & Pain Medicine*, and *Journal of Pain*. A query of the Scopus database was performed to filter the top 200 most cited articles from each journal. CY score was calculated for the top 200 articles from each journal by dividing the total number of citations by the number of years the article has been published.

**Recent Findings:**

All articles had a collective analysis of the top CY scores, the top 100 of which were further analyzed. The pain subtype, type of publication, country of origin, and senior author were extrapolated from these top 100 articles. Frequency tables were organized, revealing *Pain Journal* as the highest publishing journal out of the top 100 articles.

**Summary:**

Chronic pain was the most studied subtype of pain and narrative reviews were the most common type of evidence. Studies were also organized in five-year epochs to analyze the frequency of publications in these intervals. Results show that 2010-2014 had the highest frequency of articles published overall. Journal Impact Factor (JIF) is also an objective indicator of the average number of citations per published article from each journal. The journal with the highest JIF was Pain with an impact factor of 7.926. (6)

## Introduction

Bibliometric analysis is a statistical method used to analyze published articles, books, and citations to measure their impact within a given field. Within bibliometric analysis, citation analysis measures how often a published work is cited. This is an important tool in identifying milestone articles and areas within a given field that require more focus and exploration. Journal impact factor (JIF) is also an objective indicator of journals as a whole. JIF takes into account the average citations of articles published under them in a particular year, allowing readers and analyzers to assess a journal’s impact in its field. This provides a comparison of the quality of a journal relative to its other related journals.

Five well-known journals studying various types of pain were analyzed. These journals were the top five impact factors with regard to pain research and publications. The “Pain Physician Journal” is the official publication of the American Society of Interventional Pain Physicians (ASIPP). [1] It is a multi-disciplinary journal primarily written by and for physicians, clinicians, and scientists with an interest in interventional pain management and pain medicine. *Pain* is another major contributor to pain medicine literature. It is an open access journal that is the official publication of the International Association for the Study of Pain (IASP). *Pain* publishes literature with regards to the nature, mechanisms, and treatment of pain. [2] *Pain Medicine* is an open-access journal providing multidisciplinary research on pain and its growing need for clinical guidance and treatment. [3] It is the official journal of the American Academy of Pain Medicine (AAPM). AAPM has been working to provide educational programs and to improve clinical treatments of pain since 2011. *Regional Anesthesia & Pain Medicine* is the official publication of the American Society of Regional Anesthesia and Pain Medicine (ASRA) [4] This publication works to understand all aspects and types of pain, including acute, perioperative, transitional, and chronic. ASRA strives to address the clinical, professional, and educational needs of physicians and scientists to improve patient outcomes through research, education, and advocacy. Lastly, *Journal of Pain* is the official journal of the U.S. Association for the Study of Pain (USASP). [5] It follows ICMJE’s recommendations in its publishing of medical journals. The *Journal of Pain* focuses on publishing articles relating to all aspects of pain, including but not limited to basic, translational and clinical research, epidemiology, education, and health policy. All the above stated journals publish peer-reviewed articles with their editorial boards posted on each of their respective websites.

The impact factor of the journals are as followed: Pain- 7.926, Pain Physician- 4.396, Pain Medicine-3.637, Regional Anesthesia & Pain Medicine- 5.564, Journal of Pain- 5.383. [6] Our aim was to determine the scope and nature of the above journals’ impacts on pain literature. We conducted a bibliometric analysis of the top 100 most cited articles published in the journal since as early as 1975. By categorizing each article by type of study and pain subspecialty, we evaluated the trends in these journals’ most impactful articles over five-year epochs from 1975–2023.

## Methods

A query of the Scopus database was conducted for the top 200 articles from each journal by total citation number. [9] All articles published in their respective journals have been included since 1975.

For all 200 articles from each journal, the total number of citations was divided by the total number of years in publication to find citations per year (CY score). All articles were organized in descending order. The top 100 highest CY scoring articles are now regarded as the top 100 most cited articles. The CY rank was then assigned by organizing the studies in descending order based on the CY score from 1–100. Once this combined list was formed, further analysis was performed. Date of publication, senior author and country of origin were extracted from each article. Studies were additionally categorized into one of the following study types: Case Report, Case Series, Basic Science, Randomized Controlled Trial, Systematic Review, and Meta-Analysis. [7] Further study subtypes were added to include multiple study types if they did not fit these allocated categories. If a study fit under two study types, the one with the least frequency was used. Each article was also categorized into one of the following subspecialties: Back Pain, Chronic Pain, Postoperative Pain (Post Op Pain), Injury Pain (Pathologic pain), Fibromyalgia, and Other. [8] Analysis required further inclusion of other pain subtypes, including neuropathic pain, opioid use and pain medications, pain scales, and more. Articles with up to three incidences of a pain subtype that could not be categorized under the initial five pain subspecialties were included in the “other” category.

A panel of three co-authors were convened to categorize each study. The 100 articles were organized into five-year epochs. Trends in the type of study and pain subspecialty were found from 1975–2023. Frequency of each pain subspecialty, types of evidence, country of origin, senior author, and the journal from which an article was published were organized into frequency tables and graphs.

## Results

Srinivasa Raja et al. authored the most cited article with the highest CY rank in 2020 titled “The revised International Association for the Study of Pain definition of pain: concepts, challenges, and compromises.” It was cited 1356 times (Table 1). It came from the journal *Pain* and reevaluated the current definition of pain from the IASP Presidential Task Force.

**Table 1:** Top 100 Articles. Top 100 most cited articles were ranked by number of times cited with CY rank

CY Rank	Article Citation	Total Citations
1	Raja SN, Carr DB, Cohen M, et al. The revised International Association for the Study of Pain definition of pain: concepts, challenges, and compromises. Pain. 2020;161(9):1976–1982. 10.1097/j.pain.0000000000001939	1356
2	Treede RD, Rief W, Barke A, et al. Chronic pain as a symptom or a disease: the IASP Classification of Chronic Pain for the International Classification of Diseases (ICD-11). Pain. 2019;160(1):19–27. 10.1097/j.pain.0000000000001384	1246
3	Woolf CJ. Central sensitization: implications for the diagnosis and treatment of pain. Pain. 2011;152(3 Suppl):S2-S15. 10.1016/j.pain.2010.09.030	2867
4	Treede RD, Rief W, Barke A, et al. A classification of chronic pain for ICD-11. Pain. 2015;156(6):1003–1007. 10.1097/j.pain.0000000000000160	1455
5	Farrar JT, Young JP Jr, LaMoreaux L, Werth JL, Poole MR. Clinical importance of changes in chronic pain intensity measured on an 11-point numerical pain rating scale. Pain. 2001;94(2):149–158. 10.1016/S0304-3959(01)00349-9	3999
6	Zimmermann M. Ethical guidelines for investigations of experimental pain in conscious animals. Pain. 1983;16(2):109–110. 10.1016/0304-3959(83)90201	6991
7	Dworkin RH, Turk DC, Farrar JT, et al. Core outcome measures for chronic pain clinical trials: IMMPACT recommendations. Pain. 2005;113(1–2):9–19. 10.1016/j.pain.2004.09.012	2790
8	Vlaeyen JWS, Linton SJ. Fear-avoidance and its consequences in chronic musculoskeletal pain: a state of the art. Pain. 2000;85(3):317–332. 10.1016/S0304-3959(99)00242-0	3348
9	Bennett GJ, Xie YK. A peripheral mononeuropathy in rat that produces disorders of pain sensation like those seen in man. Pain. 1988;33(1):87–107. 10.1016/0304-3959(88)90209-6	4610
10	Nicholas M, Vlaeyen JWS, Rief W, et al. The IASP classification of chronic pain for ICD-11: chronic primary pain. Pain. 2019;160(1):28–37. 10.1097/j.pain.0000000000001390	514
11	Hargreaves K, Dubner R, Brown F, Flores C, Joris J. A new and sensitive method for measuring thermal nociception in cutaneous hyperalgesia. Pain. 1988;32(1):77–88. 10.1016/0304-3959(88)90026-7	4423
12	Scholz J, Finnerup NB, Attal N, et al. The IASP classification of chronic pain for ICD-11: chronic neuropathic pain. Pain. 2019;160(1):53–59. 10.1097/j.pain.0000000000001365	460
13	Melzack R. The McGill Pain Questionnaire: major properties and scoring methods. Pain. 1975;1(3):277–299. 10.1016/0304-3959(75)90044-5	5452
14	van Hecke O, Austin SK, Khan RA, Smith BH, Torrance N. Neuropathic pain in the general population: a systematic review of epidemiological studies [published correction appears in Pain. 2014 Sep;155(9):1907]. Pain. 2014;155(4):654–662. 10.1016/j.pain.2013.11.013	989
15	Dworkin RH, O'Connor AB, Backonja M, et al. Pharmacologic management of neuropathic pain: evidence-based recommendations. Pain. 2007;132(3):237–251. 10.1016/j.pain.2007.08.033	1714
16	Rolke R, Baron R, Maier C, et al. Quantitative sensory testing in the German Research Network on Neuropathic Pain (DFNS): standardized protocol and reference values [published correction appears in Pain. 2006 Nov;125(1–2):197]. Pain. 2006;123(3):231–243. 10.1016/j.pain.2006.01.041	1778
17	Vowles KE, McEntee ML, Julnes PS, Frohe T, Ney JP, van der Goes DN. Rates of opioid misuse, abuse, and addiction in chronic pain: a systematic review and data synthesis. Pain. 2015;156(4):569–576. 10.1097/01.j.pain.0000460357.01998.f1	820
18	Benyamin R, Trescot AM, Datta S, et al. Opioid complications and side effects. Pain Physician. 2008;11(2 Suppl):S105-S120	1527
19	Finnerup NB, Haroutounian S, Kamerman P, et al. Neuropathic pain: an updated grading system for research and clinical practice. Pain. 2016;157(8):1599–1606. 10.1097/j.pain.0000000000000492	702
20	Bouhassira D, Attal N, Alchaar H, et al. Comparison of pain syndromes associated with nervous or somatic lesions and development of a new neuropathic pain diagnostic questionnaire (DN4). Pain. 2005;114(1–2):29–36. 10.1016/j.pain.2004.12.010	1805
21	Melzack R. The short-form McGill Pain Questionnaire. Pain. 1987;30(2):191–197. 10.1016/0304-3959(87)91074-8	3417
22	Seretny M, Currie GL, Sena ES, et al. Incidence, prevalence, and predictors of chemotherapy-induced peripheral neuropathy: A systematic review and meta-analysis. Pain. 2014;155(12):2461–2470. 10.1016/j.pain.2014.09.020	853
23	King S, Chambers CT, Huguet A, et al. The epidemiology of chronic pain in children and adolescents revisited: a systematic review. Pain. 2011;152(12):2729–2738. 10.1016/j.pain.2011.07.016	1123
24	Ferreira-Valente MA, Pais-Ribeiro JL, Jensen MP. Validity of four pain intensity rating scales. Pain. 2011;152(10):2399–2404. 10.1016/j.pain.2011.07.005	1114
25	Ho Kim S, Mo Chung J. An experimental model for peripheral neuropathy produced by segmental spinal nerve ligation in the rat. Pain. 1992;50(3):355–363. 10.1016/0304-3959(92)90041-9	2718
26	Manchikanti L, Helm S 2nd, Fellows B, et al. Opioid epidemic in the United States. Pain Physician. 2012;15(3 Suppl):ES9-ES38	961
27	Bouhassira D, Lantéri-Minet M, Attal N, Laurent B, Touboul C. Prevalence of chronic pain with neuropathic characteristics in the general population. Pain. 2008;136(3):380–387. 10.1016/j.pain.2007.08.013	1221
28	Ji RR, Berta T, Nedergaard M. Glia and pain: is chronic pain a gliopathy?. Pain. 2013;154 Suppl 1(0 1):S10-S28. 10.1016/j.pain.2013.06.022	814
29	Jensen TS, Baron R, Haanpää M, et al. A new definition of neuropathic pain. Pain. 2011;152(10):2204–2205. 10.1016/j.pain.2011.06.017	970
30	Waddell G, Newton M, Henderson I, Somerville D, Main CJ. A Fear-Avoidance Beliefs Questionnaire (FABQ) and the role of fear-avoidance beliefs in chronic low back pain and disability. Pain. 1993;52(2):157–168. 10.1016/0304-3959(93)90127-B	2359
31	Von Korff M, Ormel J, Keefe FJ, Dworkin SF. Grading the severity of chronic pain. Pain. 1992;50(2):133–149. 10.1016/0304-3959(92)90154-4	2312
32	Decosterd I, Woolf CJ. Spared nerve injury: an animal model of persistent peripheral neuropathic pain. Pain. 2000;87(2):149–158. 10.1016/S0304-3959(00)00276-1	1679
33	Price DD, McGrath PA, Rafii A, Buckingham B. The validation of visual analogue scales as ratio scale measures for chronic and experimental pain. Pain. 1983;17(1):45–56. 10.1016/0304-3959(83)90126-4	2772
34	Haanpää M, Attal N, Backonja M, et al. NeuPSIG guidelines on neuropathic pain assessment. Pain. 2011;152(1):14–27. 10.1016/j.pain.2010.07.031	821
35	Tjølsen A, Berge OG, Hunskaar S, Rosland JH, Hole K. The formalin test: an evaluation of the method. Pain. 1992;51(1):5–17. 10.1016/0304-3959(92)90003-T	2034
36	Finnerup NB, Sindrup SH, Jensen TS. The evidence for pharmacological treatment of neuropathic pain. Pain. 2010;150(3):573–581. 10.1016/j.pain.2010.06.019	841
37	Finnerup NB, Otto M, McQuay HJ, Jensen TS, Sindrup SH. Algorithm for neuropathic pain treatment: an evidence based proposal. Pain. 2005;118(3):289–305. 10.1016/j.pain.2005.08.013	1156
38	Hicks CL, von Baeyer CL, Spafford PA, van Korlaar I, Goodenough B. The Faces Pain Scale-Revised: toward a common metric in pediatric pain measurement. Pain. 2001;93(2):173–183. 10.1016/S0304-3959(01)00314-1	1405
39	Lee M, Silverman SM, Hansen H, Patel VB, Manchikanti L. A comprehensive review of opioid-induced hyperalgesia. Pain Physician. 2011;14(2):145–161	759
40	Jensen MP, Karoly P, Braver S. The measurement of clinical pain intensity: a comparison of six methods. Pain. 1986;27(1):117–126. 10.1016/0304-3959(86)90228-9	2307
41	Vlaeyen JWS, Kole-Snijders AMJ, Boeren RGB, van Eek H. Fear of movement/(re)injury in chronic low back pain and its relation to behavioral performance. Pain. 1995;62(3):363–372. 10.1016/0304-3959(94)00279-N	1732
42	Harden NR, Bruehl S, Perez RSGM, et al. Validation of proposed diagnostic criteria (the "Budapest Criteria") for Complex Regional Pain Syndrome. Pain. 2010;150(2):268–274. 10.1016/j.pain.2010.04.030	797
43	Coderre TJ, Katz J, Vaccarino AL, Melzack R. Contribution of central neuroplasticity to pathological pain: review of clinical and experimental evidence. Pain. 1993;52(3):259–285. 10.1016/0304-3959(93)90161-H	1795
44	Vlaeyen JWS, Linton SJ. Fear-avoidance model of chronic musculoskeletal pain: 12 years on. Pain. 2012;153(6):1144–1147. 10.1016/j.pain.2011.12.009	656
45	Arendt-Nielsen L, Nie H, Laursen MB, et al. Sensitization in patients with painful knee osteoarthritis. Pain. 2010;149(3):573–581. 10.1016/j.pain.2010.04.003	763
46	Atluri S, Manchikanti L, Hirsch JA. Expanded Umbilical Cord Mesenchymal Stem Cells (UC-MSCs) as a Therapeutic Strategy in Managing Critically Ill COVID-19 Patients: The Case for Compassionate Use. Pain Physician. 2020;23(2):E71-E83	176
47	Noseda R, Burstein R. Migraine pathophysiology: anatomy of the trigeminovascular pathway and associated neurological symptoms, cortical spreading depression, sensitization, and modulation of pain. Pain. 2013;154 Suppl 1:S44-S53. 10.1016/j.pain.2013.07.021	568
48	Maier C, Baron R, Tölle TR, et al. Quantitative sensory testing in the German Research Network on Neuropathic Pain (DFNS): somatosensory abnormalities in 1236 patients with different neuropathic pain syndromes. Pain. 2010;150(3):439–450. 10.1016/j.pain.2010.05.002	727
49	Hodges PW, Tucker K. Moving differently in pain: a new theory to explain the adaptation to pain. Pain. 2011;152(3 Suppl):S90-S98. 10.1016/j.pain.2010.10.020	666
50	Kalso E, Edwards JE, Moore AR, McQuay HJ. Opioids in chronic non-cancer pain: systematic review of efficacy and safety. Pain. 2004;112(3):372–380. 10.1016/j.pain.2004.09.019	1049
51	Manchikanti L, Kaye AM, Knezevic NN, et al. Responsible, Safe, and Effective Prescription of Opioids for Chronic Non-Cancer Pain: American Society of Interventional Pain Physicians (ASIPP) Guidelines. Pain Physician. 2017;20(2S):S3-S92	321
52	Amtmann D, Cook KF, Jensen MP, et al. Development of a PROMIS item bank to measure pain interference. Pain. 2010;150(1):173–182. 10.1016/j.pain.2010.04.025	695
53	Woolf CJ, Thompson SWN. The induction and maintenance of central sensitization is dependent on N-methyl-D-aspartic acid receptor activation; implications for the treatment of post-injury pain hypersensitivity states. Pain. 1991;44(3):293–299. 10.1016/0304-3959(91)90100-C	1699
54	Morley S, Eccleston C, Williams A. Systematic review and meta-analysis of randomized controlled trials of cognitive behaviour therapy and behaviour therapy for chronic pain in adults, excluding headache. Pain. 1999;80(1–2):1–13. 10.1016/s0304-3959(98)00255-3	1264
55	Kumar K, Taylor RS, Jacques L, et al. Spinal cord stimulation versus conventional medical management for neuropathic pain: a multicentre randomised controlled trial in patients with failed back surgery syndrome. Pain. 2007;132(1–2):179–188. 10.1016/j.pain.2007.07.028	836
56	Loeser JD, Treede RD. The Kyoto protocol of IASP Basic Pain Terminology. Pain. 2008;137(3):473–477. 10.1016/j.pain.2008.04.025	783
57	Crombez G, Vlaeyen JW, Heuts PH, Lysens R. Pain-related fear is more disabling than pain itself: evidence on the role of pain-related fear in chronic back pain disability. Pain. 1999;80(1–2):329–339. 10.1016/s0304-3959(98)00229-2	1243
58	Hunskaar S, Hole K. The formalin test in mice: dissociation between inflammatory and non-inflammatory pain. Pain. 1987;30(1):103–114. 10.1016/0304-3959(87)90088-1	1830
59	Turk DC, Dworkin RH, Allen RR, et al. Core outcome domains for chronic pain clinical trials: IMMPACT recommendations. Pain. 2003;106(3):337–345. 10.1016/j.pain.2003.08.001	1001
60	Bouhassira D, Attal N, Fermanian J, et al. Development and validation of the Neuropathic Pain Symptom Inventory. Pain. 2004;108(3):248–257. 10.1016/j.pain.2003.12.024	943
61	Maniadakis N, Gray A. The economic burden of back pain in the UK. Pain. 2000;84(1):95–103. 10.1016/S0304-3959(99)00187-6	1140
62	Kerns RD, Turk DC, Rudy TE. The West Haven-Yale Multidimensional Pain Inventory (WHYMPI). Pain. 1985;23(4):345–356. 10.1016/0304-3959(85)90004-1	1870
63	Dubuisson D, Dennis SG. The formalin test: a quantitative study of the analgesic effects of morphine, meperidine, and brain stem stimulation in rats and cats. Pain. 1977;4(2):161–174. 10.1016/0304-3959(77)90130-0	2233
64	Patel KV, Guralnik JM, Dansie EJ, Turk DC. Prevalence and impact of pain among older adults in the United States: findings from the 2011 National Health and Aging Trends Study. Pain. 2013;154(12):2649–2657. 10.1016/j.pain.2013.07.029	485
65	Rosenstiel AK, Keefe FJ. The use of coping strategies in chronic low back pain patients: relationship to patient characteristics and current adjustment. Pain. 1983;17(1):33–44. 10.1016/0304-3959(83)90125-2	1935
66	Seltzer Z, Dubner R, Shir Y. A novel behavioral model of neuropathic pain disorders produced in rats by partial sciatic nerve injury. Pain. 1990;43(2):205–218. 10.1016/0304-3959(90)91074-S	1589
67	Greenspan JD, Craft RM, LeResche L, et al. Studying sex and gender differences in pain and analgesia: a consensus report. Pain. 2007;132 Suppl 1(Suppl 1):S26-S45. 10.1016/j.pain.2007.10.014	758
68	Trescot AM, Datta S, Lee M, Hansen H. Opioid pharmacology. Pain Physician. 2008;11(2 Suppl):S133-S153	700
69	Cohen SP, Baber ZB, Buvanendran A, et al. Pain Management Best Practices from Multispecialty Organizations During the COVID-19 Pandemic and Public Health Crises. Pain Med. 2020;21(7):1331–1346. 10.1093/pm/pnaa127	139
70	Collins SL, Moore RA, McQuay HJ. The visual analogue pain intensity scale: what is moderate pain in millimetres?. Pain. 1997;72(1–2):95–97. 10.1016/s0304-3959(97)00005-5	1203
71	Manchikanti L, Abdi S, Atluri S, et al. An update of comprehensive evidence-based guidelines for interventional techniques in chronic spinal pain. Part II: guidance and recommendations. Pain Physician. 2013;16(2 Suppl):S49-S283	459
72	Green CR, Anderson KO, Baker TA, et al. The unequal burden of pain: confronting racial and ethnic disparities in pain [published correction appears in Pain Med. 2005 Jan-Feb;6(1):99. Kaloukalani, Donna A [corrected to Kalauokalani, Donna A]]. Pain Med. 2003;4(3):277–294. 10.1046/j.1526-4637.2003.03034.x	910
73	Webster LR, Webster RM. Predicting aberrant behaviors in opioid-treated patients: preliminary validation of the Opioid Risk Tool. Pain Med. 2005;6(6):432–442. 10.1111/j.1526-4637.2005.00072.x	817
74	Sindrup SH, Jensen TS. Efficacy of pharmacological treatments of neuropathic pain: an update and effect related to mechanism of drug action. Pain. 1999;83(3):389–400. 10.1016/S0304-3959(99)00154-2	1085
75	Apkarian VA, Hashmi JA, Baliki MN. Pain and the brain: specificity and plasticity of the brain in clinical chronic pain. Pain. 2011;152(3 Suppl):S49-S64. 10.1016/j.pain.2010.11.010	540
76	Veehof MM, Oskam MJ, Schreurs KMG, Bohlmeijer ET. Acceptance-based interventions for the treatment of chronic pain: a systematic review and meta-analysis. Pain. 2011;152(3):533–542. 10.1016/j.pain.2010.11.002	533
77	Unruh AM. Gender variations in clinical pain experience. Pain. 1996;65(2–3):123–167. 10.1016/0304-3959(95)00214-6	1193
78	Serlin RC, Mendoza TR, Nakamura Y, Edwards KR, Cleeland CS. When is cancer pain mild, moderate or severe? Grading pain severity by its interference with function. Pain. 1995;61(2):277–284. 10.1016/0304-3959(94)00178-H	1228
79	McCracken LM, Vowles KE, Eccleston C. Acceptance of chronic pain: component analysis and a revised assessment method. Pain. 2004;107(1–2):159–166. 10.1016/j.pain.2003.10.012	822
80	Racine M, Tousignant-Laflamme Y, Kloda LA, Dion D, Dupuis G, Choinière M. A systematic literature review of 10 years of research on sex/gender and experimental pain perception—part 1: are there really differences between women and men?. Pain. 2012;153(3):602–618. 10.1016/j.pain.2011.11.025	475
81	Harden RN, Bruehl S, Stanton-Hicks M, Wilson PR. Proposed new diagnostic criteria for complex regional pain syndrome. Pain Med. 2007;8(4):326–331. 10.1111/j.1526-4637.2006.00169.x	688
82	Picavet HS, Schouten JS. Musculoskeletal pain in the Netherlands: prevalences, consequences and risk groups, the DMC(3)-study. Pain. 2003;102(1–2):167–178. 10.1016/s0304-3959(02)00372-x	853
83	Goldstein DJ, Lu Y, Detke MJ, Lee TC, Iyengar S. Duloxetine vs. placebo in patients with painful diabetic neuropathy. Pain. 2005;116(1–2):109–118. 10.1016/j.pain.2005.03.029	763
84	Birnbaum HG, White AG, Schiller M, Waldman T, Cleveland JM, Roland CL. Societal costs of prescription opioid abuse, dependence, and misuse in the United States. Pain Med. 2011;12(4):657–667. 10.1111/j.1526-4637.2011.01075.x	506
85	Shah S, Diwan S, Kohan L, et al. The Technological Impact of COVID-19 on the Future of Education and Health Care Delivery. Pain Physician. 2020;23(4S):S367-S380	126
86	Jensen MP, Turner JA, Romano JM, Fisher LD. Comparative reliability and validity of chronic pain intensity measures. Pain. 1999;83(2):157–162. 10.1016/s0304-3959(99)00101-3	999
87	Manchikanti L, Singh V, Datta S, Cohen SP, Hirsch JA; American Society of Interventional Pain Physicians. Comprehensive review of epidemiology, scope, and impact of spinal pain. Pain Physician. 2009;12(4):E35-E70	581
88	Bates D, Schultheis BC, Hanes MC, et al. A Comprehensive Algorithm for Management of Neuropathic Pain [published correction appears in Pain Med. 2023 Feb 1;24(2):219]. Pain Med. 2019;20(Suppl 1):S2-S12. 10.1093/pm/pnz075	165
89	Yarnitsky D, Crispel Y, Eisenberg E, et al. Prediction of chronic post-operative pain: pre-operative DNIC testing identifies patients at risk. Pain. 2008;138(1):22–28. 10.1016/j.pain.2007.10.033	607
90	Wylde V, Hewlett S, Learmonth ID, Dieppe P. Persistent pain after joint replacement: prevalence, sensory qualities, and postoperative determinants. Pain. 2011;152(3):566–572. 10.1016/j.pain.2010.11.023	473
91	Pan M, Li Q, Li S, et al. Percutaneous Endoscopic Lumbar Discectomy: Indications and Complications. Pain Physician. 2020;23(1):49–56	118
92	Bennett M. The LANSS Pain Scale: the Leeds assessment of neuropathic symptoms and signs. Pain. 2001;92(1–2):147–157. 10.1016/s0304-3959(00)00482-6	863
93	de Mos M, de Bruijn AG, Huygen FJ, Dieleman JP, Stricker BH, Sturkenboom MC. The incidence of complex regional pain syndrome: a population-based study. Pain. 2007;129(1–2):12–20. 10.1016/j.pain.2006.09.008	622
94	Blyth FM, March LM, Brnabic AJ, Jorm LR, Williamson M, Cousins MJ. Chronic pain in Australia: a prevalence study. Pain. 2001;89(2–3):127–134. 10.1016/s0304-3959(00)00355-9	837
95	Scott J, Huskisson EC. Graphic representation of pain. Pain. 1976;2(2):175–184	1774
96	Perquin CW, Hazebroek-Kampschreur AAJM, Hunfeld JAM, et al. Pain in children and adolescents: a common experience. Pain. 2000;87(1):51–58. 10.1016/S0304-3959(00)00269-4	867
97	Chessell IP, Hatcher JP, Bountra C, et al. Disruption of the P2X7 purinoceptor gene abolishes chronic inflammatory and neuropathic pain. Pain. 2005;114(3):386–396. 10.1016/j.pain.2005.01.002	675
98	Treede RD, Kenshalo DR, Gracely RH, Jones AK. The cortical representation of pain. Pain. 1999;79(2–3):105–111. 10.1016/s0304-3959(98)00184-5	879
99	Zhuang ZY, Gerner P, Woolf CJ, Ji RR. ERK is sequentially activated in neurons, microglia, and astrocytes by spinal nerve ligation and contributes to mechanical allodynia in this neuropathic pain model. Pain. 2005;114(1–2):149–159. 10.1016/j.pain.2004.12.022	659
100	Harden RN, Oaklander AL, Burton AW, et al. Complex regional pain syndrome: practical diagnostic and treatment guidelines, 4th edition. Pain Med. 2013;14(2):180–229. 10.1111/pme.12033	366

Of the five journals queried (*Pain, Pain Physician, Pain Medicine, Regional Anesthesia and Pain Medicine*, and *Journal of Pain*), *Pain* had the most articles in the top 100 CY ranked articles, with 83 total. Pain Physician and Pain Medicine followed with ten and seven articles, respectively. Regional Anesthesia and Pain Medicine and the Journal of Pain did not have articles in the top 100 regarding calculated CY scores (Table 2).

**Table 2:** Journal frequency. Frequency of journals from top 100 articles

Journal Name	Frequency	Impact Factor (Past 3 years)
Pain	83	5.479
Pain Physician	11	4.204
Pain Medicine	6	2.241
Regional Anesthesia and Pain Medicine	0	3.155
Journal of Pain	0	4.937

The top 100 articles featured 83 distinct senior authors extrapolated from article citations. The most common senior author was Dr. Laxmaiah Manchikanti (Pain Management Center of Paducah), who wrote four articles, followed by Dr. Rolf-Detlef Treede (Mannheim Center for Translational Neuroscience). Dr. Johan W.S. Vlaeyen, Dr. Nanna Finnerup, Dr. Didier Bouhassira, and Dr. Robert N. Harden each had three articles that were attributed to senior authors. Dr. Clifford J. Woolf, Dr. Robert H. Dworkin, Dr. Ronald Melzack, and Dr. Mark P. Jensen were the senior authors of two articles. The remainder of the authors had one article in our analysis (Table 3).

**Table 3:** Senior authors ranked based on frequency of studies published in the top 100 most cited articles

Frequency	Senior Author Name
4	Laxmaiah Manchikanti
3	Rolf-Detlef Treede
3	Johan W.S. Vlaeyen
3	Nanna B Finnerup
3	Didier Bouhassira
3	Norman R. Harden
2	Clifford J. Woolf
2	Robert H. Dworkin
2	Ronald Melzack
2	Mark P. Jensen
1	Srinivasa Raja, John T. Farrar, Manfred Zimmermann, Gary J. Bennett, Michael Nicholas, K. Hargreaves, Joachim Scholz, O. van Hecke, R. Rolke, Kevin E. Vowles, Ramsin Benyamin, Marta Seretny, Sara King, Maria Alexandra Ferreira-Valente, Sun Ho Kim, Ru-Rong Ji, Troels S. Jensen, Gordan Waddell, Michael Von Korff, Isabelle Decosterd, Donald D. Price, Maija Haanpää, Arne Tjølsen, Carrie L. Hicks, Marion Lee, Terence J. Coderre, Lars Arendt-Nielsen, Sairam Atluri, Rodrigo Noseda, C. Maier, Paul W. Hodges, Eija Kalso, Dagmar Amtmann, S. Morley, Krishna Kumar, John D. Loeser, G. Crombez, Steinar Kunskaar, Dennis C. Turk, Nikolaos Maniadakis, Robert Kerns, David Dubuisson, Kushang Patel, Anne K. Rosenstiel, Ze'ev Seltzer, Joel D. Greenspan, Andrea M. Trescot, Steve P. Cohen, S.L. Collins, Carmen R. Green, Lynn Webster, Søren H. Sindrup, Vania A. Apkarian, Martine M. Veehof, A.M. Unruh, Ronad C. Serlin, Lance M. McCracken, Mélanie Racine, H.S.J. Picavet, David J. Goldstein, Howard G. Birnbaurn, Shalini Shah, Daniel Bates, David Yarnitsky, Vikki Wylde, Mingming Pan, M. Bennett, M. de Mos, F.M. Blyth, J. Scott, Christel W. Perquin, Iain P. Chessell, Zhi-Ye Zhuang

The country of origin of the publications were also analyzed, associated with the senior author’s affiliated program’s location. The United States had the highest frequency with 45 articles, followed by the United Kingdom [10] and Canada [8] (Table 4).

**Table 4:** Frequency of publications by country of origin

Frequency	Country of Origin
45	USA
10	United Kingdom
8	Canada
7	Netherlands
6	Germany
6	Denmark
4	Australia
3	France
2	Norway, Israel, Finland, Belgium
1	Switzerland, Portugal, China

**Fig. 1:**
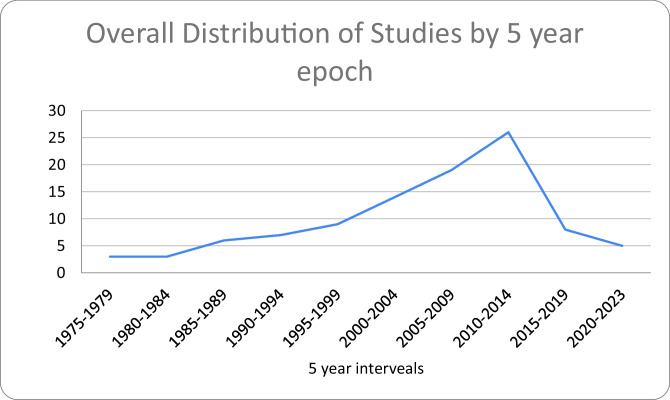
Distribution of top 100 articles in 5 year epochs

Table 5 shows the frequency of pain types. Chronic and neuropathic pain were studied the most, with 22 and 20 articles, respectively. This was followed by general pain [9] and pain medications and opioid use (6 each). Of note, all three COVID management publications began in 2020 (Fig. 1).

**Table 5:** Frequency of pain types studied in top 100 articles

Pain Type	Frequency of Pain Types
Chronic pain	22
Neuropathic pain	20
General pain	9
Pain medication	6
Opioid use	6
Post op pain	5
Pain scale	5
complex regional pain syndrome	4
Back pain	4
Pathologic pain	3
other (COVID management)	3
other (pain perception)	2
other (pain terminology, thermal nociception, peripheral neuropathy, pediatric pain, pain cost, musculoskeletal pain, migraine pain, knee pain, fear avoidance model, experimental pain, spinal pain)	1

**Fig. 2:**
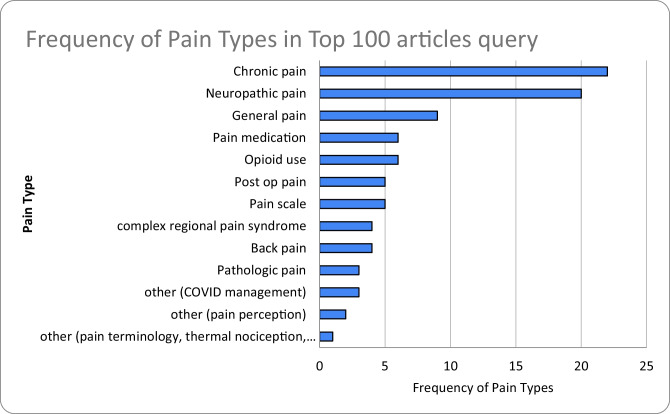
Frequency of pain subtypes studied in top 100 articles query. “Other” includes pain terminology, thermal nociception, peripheral neuropathy, pediatric pain, pain cost, musculoskeletal pain, migraine pain, knee pain, fear avoidance model, experimental pain, spinal pain

Table 6 reveals the frequency of types of evidence. The most predominant evidence type was narrative studies (40 total). Next, meta-analysis and systematic reviews had 17 articles each. Of note, prospective cohorts, case–control studies, and case reports/case series did not appear in the top 100 articles (Fig. 2).

**Table 6:** Frequency of types of evidence analyzed in top 100 articles

Type of Evidence	Frequency of Types of Evidence
Narrative Review	40
Meta-Analysis/Systematic Review	17
Experimental Trials	16
Practice Guidelines	9
Surveys	8
other (comparative study)	6
Editorials	3
Retrospective Cohort	1
Prospective Cohort	0
Case–Control Studies	0
Case Reports & Series	0

**Fig. 3:**
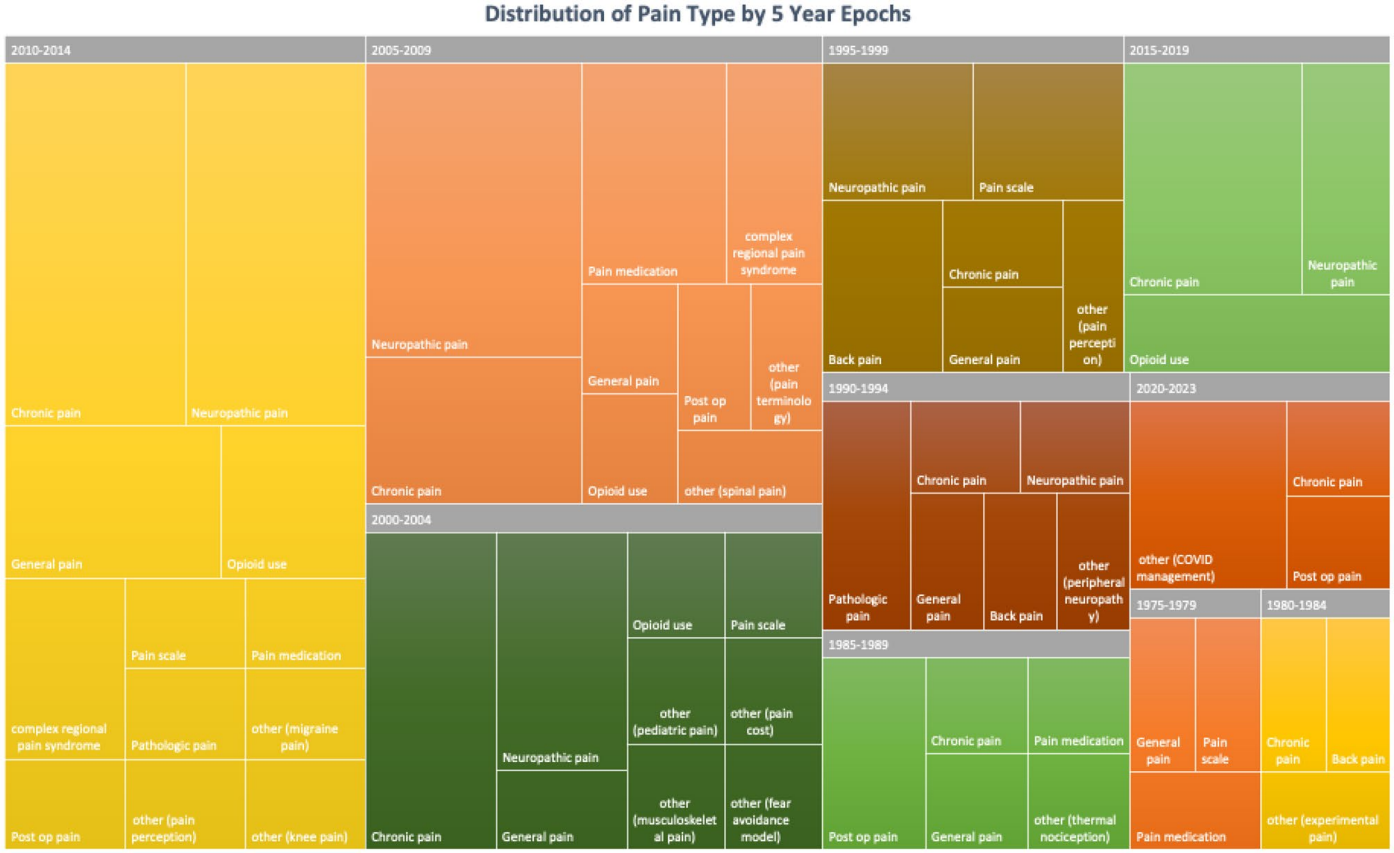
Distribution of pain subtypes in 5 year epochs based on publication year

We also observed a peak in article publications from 2010–2014, with 26 total articles in this timeframe, 6 of which were about neuropathic pain. The 2005–2009 epoch also revealed 6 studies regarding neuropathic pain. Chronic pain was most consistently studied from 1975 through 2023, with 1975–1979 being the only time range where chronic pain articles were not published in the top 100 articles from any journals noted above (Table 7). Figure 3 shows a visual chart revealing which types of pain were studied in their respective epochs.

**Table 7:** Frequency of articles organized by publication year in 5 year epochs

Year Epoch	Frequency of Articles
1975–1979	3
1980–1984	3
1985–1989	6
1990–1994	7
1995–1999	9
2000–2004	14
2005–2009	19
2010–2014	26
2015–2019	8
2020–2023	5

## Discussion

*The Pain Physician*, *Pain*, *Pain Medicine*, *Regional Anesthesia and Pain Medicine,* and *Journal of Pain* are peer-reviewed journals invested in understanding and developing new clinical practices to alleviate the elevated economic burden of pain today, namely chronic pain. This is confirmed by more literature showing an increase in the prevalence of chronic pain among United States patients. [10] (Table 8).

**Table 8:** Frequency of pain subtypes organized by publication year in 5 year epochs

Number of Articles	Year Range	Pain Type	Number of Studies
3	1975–1979	General Pain	1
		Pain Scale	1
		Pain Medication	1
3	1980–1984	Chronic Pain	1
		Back Pain	1
		other (experimental pain)	1
6	1985–1989	Chronic Pain	1
		General Pain	1
		Post op Pain	2
		Pain Medication	1
		Other (thermal nociception)	1
7	1990–1994	Chronic Pain	1
		Neuropathic Pain	1
		General Pain	1
		Back Pain	1
		Pathologic Pain	2
		Other (peripheral neuropathy)	1
9	1995–1999	Chronic Pain	1
		Neuropathic Pain	2
		General Pain	1
		Pain Scale	2
		Back Pain	2
		other (pain perception)	1
14	2000–2004	Chronic Pain	4
		Neuropathic Pain	3
		General Pain	1
		Opioid use	1
		Pain Scale	1
		other (pediatric pain)	1
		other (pain cost)	1
		other (musculoskeletal pain)	1
		other (fear avoidance model)	1
19	2005–2009	Chronic Pain	3
		Neuropathic Pain	6
		General Pain	1
		Opioid use	1
		Post op Pain	1
		Pain Medication	3
		Complex regional pain syndrome	2
		other (pain terminology)	1
		other (spinal pain)	1
26	2010–2014	Chronic Pain	6
		Neuropathic Pain	6
		General Pain	3
		Opioid use	2
		Post op Pain	1
		Pain Scale	1
		Pain Medication	1
		Complex regional pain syndrome	2
		Pathologic Pain	1
		other (pain perception)	1
		other (migraine pain)	1
		other (knee pain)	1
8	2015–2019	Chronic Pain	4
		Neuropathic Pain	2
		Opioid use	2
5	2020–2023	Chronic Pain	1
		Post op Pain	1
		other (COVID management)	3

The *Pain Physician* is a journal with over two decades of publications and 6 issues per year since 2007 and has a journal impact factor of 4.396. Pain Physician is the official publication of the American Society of Interventional Pain Physicians (ASIPP). *Pain* is another journal publishing since 1975. It is the official publication of the International Association for the Study of Pain (IASP). *Pain* has a journal impact factor of 7.926. *Pain Medicine* has been publishing journals since 2000 as the official publication of the American Academy of Pain Medicine (AAPM). This journal has an impact factor of 3.637. *Regional Anesthesia and Pain Medicine* (RAPM), with a journal impact factor of 5.564 is the official publication for the American Society of Regional Anesthesia and Pain Medicine (ASRA). Lastly, *Journal of Pain*, the official journal of the U.S. Association for the Study of Pain (USASP), has an impact factor of 5.383. All of the above journals are open access with their main objectives to continue the pursuit in knowledge and treatment of various types of pain.

Citation analysis was also performed to compare articles from these five journals based on their CY scores. Of note, articles that had the highest overall citation number may not have been placed highest concerning CY score. If an article had a higher citation count over a shorter period, this would boost the CY score and, therefore have it higher on our CY ranking list.

Our first CY ranked article was titled “The revised International Association for the Study of Pain definition of pain: concepts, challenges, and compromises.” It was cited 1356 times and was released in 2020 with a CY score of 4452. It was published within *Pain*. The senior author is Dr. Srinivasa Raja, MD. He is a professor of Anesthesiology and Neurology and the Director of Pain research at Johns Hopkins University School of Medicine. His interests lie in the management of chronic neuropathic pain states and he recently put his work into understanding the peripheral and central mechanisms of neuropathic pain. The article is a review that provides dialogue and discussions of the revised definitions of pain according to the IASP council. The current definition of pain at the time of publishing was “An unpleasant sensory and emotional experience associated with actual or potential tissue damage or described in terms of such damage.” The new revision is “An unpleasant sensory and emotional experience associated with, or resembling that associated with actual or potential tissue damage.” The task force used multiple models, including monthly web-web discussions, and in-person meetings to arrive at the new pain criteria. The public consultation was also acknowledged. 808 responses from 46 countries, including clinicians, clinical and basic science researchers, administrators, educators or trainees/students, were submitted. [11•].

The second most cited article was “Central sensitization: Implications for the diagnosis and treatment of pain”. It had 2867 total citations and was published in 2019. It had a CY score of 238.9, making it third in our CY rank. It was also published with *Pain* in 2011. Published by Dr. Clifford J. Woolf, M.B., B. Ch., PhD, he is affiliated with F.M. Kirby Neurobiology Center and the Neurobiology program at Boston Children’s Hospital. His research interests are investigating the functional, chemical, and structural plasticity of neurons that contribute to adaptive and maladaptive changes, focusing specifically on pain in the injured and neurodegenerative conditions. In the article, Dr. Woolf discusses central sensitization, its history and its role in neurodegenerative pathologies such as rheumatoid arthritis, osteoarthritis, temporomandibular disorders, fibromyalgia and more. He describes this pain as being caused by central sensitization due to the “increased state of excitability”. He states there is much more to be learned about central sensitization including genetic and molecular mechanisms that may contribute to chronic pain in some individuals. [12].

The article with the second CY rank is “Chronic pain as a symptom or a disease: the IASP Classification of Chronic Pain for the International Classification of Diseases (ICD-11)”. It had 1246 total citations and was published in 2019 with a CY score of 311.5. The senior author is Dr. Rolf-Detlef Treede MD, PhD, Chair of Neurophysiology at the Mannheim Center for Translational Neuroscience in Mannheim, Germany. Dr. Treede specifically researches damaging stimuli to the nervous system and its relationship to acute and chronic pain. He also works to develop tools to assess nociceptive pathways. In this article, Dr. Treede et al*.* presents a systematic classification of chronic pain that is part of the ICD-11 chronic pain diagnosis. This classification was lacking in ICD, causing significant confusion in treatment pathways for chronic pain patients. In collaboration with IASP and the World Health Organization (WHO), a new classification system was created to apply for specialized pain management. Chronic pain now serves as the “parent code” for seven other codes that are made up of the most common clinical pain conditions, including chronic primary pain, chronic cancer-related pain, chronic postsurgical or posttraumatic pain, chronic neuropathic pain, chronic secondary headache or orofacial pain, chronic secondary visceral pain and chronic secondary musculoskeletal pain. [13•].

The most cited article was at CY rank 13 with 5452 citations. Published in 1975 through *Pain*, titled “The McGill Pain Questionnaire: Major properties and scoring methods”. Authored by Dr. Ronald Melzack, PhD, Canadian psychologist and professor of psychology at McGill University, he introduces the McGill Pain Questionnaire. In it, he describes how to use the questionnaire and what to gather from the results. His three main measures are the pain rating index, number of words chosen and present pain intensity. Dr. Melzack also provides analysis of a research project that uses the McGill Questionnaire, revealing that the pain rating index was an objective method to report differences in pain with various treatments in research. [14].

Our analysis found a total of 83 senior authors contributing to the top 100 most cited articles published across all 5 journals. The most published senior author was Dr. Laxmaiah Manchikanti, MD. Dr. Manchikanti founded the American Society of Interventional Pain Physicians (ASIPP), the Society of Interventional Pain Management Surgery Centers (SIPMS) and the *Pain Physician*. Dr. Rolf-Detlef Treede, Dr. Johan W.S. Vlaeyen, Dr. Nanna Finnerup, Dr. Didier Bouhassira, and Dr. Robert N. Harden each had 3 articles. Combined, these senior authors contributed to 19 total articles.

There are five types of scientific evidence: case reports/case series, case–control studies, cohort studies, randomized control trials and systematic reviews. However, with the number of articles which could not be categorized under these evidence types, new study types had to be used in addition. 40 of the articles were narrative reviews, which included studies such as basic science, review articles and literature reviews. 17 articles were meta-analysis and systematic reviews, followed by 16 experimental trial articles. Experimental trials included experimental or observational studies, clinical trials, and randomized control trials. Practice guidelines included between-subject and scale development studies (9 total). There were 8 survey studies which included prevalence studies. Comparative studies had 6 total articles. 3 articles were editorials and 1 article was a retrospective cohort. There were no prospective cohort, case–control or case-reports/case series studies. With the increasing prevalence of pain by almost 10% from 2002–2018, the need to understand the multidimensional aspects becomes vital for improving care and reducing healthcare costs. [15] The escalating prevalence and amount of information on these two types of pain states makes it increasingly difficult to keep up with all the data provided. To alleviate this burden on the scientific community, authors have and continue to compile original research articles to analyze, to summarize, and to critique them in newly synthesized review articles. [16] As expected, most of the top 100 cited articles were Level I and II studies, which indicates the strength of the evidence provided with Level I being based on strong experimental designs and studies. Examples can include RCT’s, systematic reviews of RCT’s and can include meta-analyses. Level V is the opposite end of the scale and is based on experiences and non-research evidence. While useful, Level V evidence should not be completely ignored just as Level I evidence should not be considered entirely generalized. [17, 18].

With the growing prevalence of chronic pain and back pain, a predictable increase in the number of articles detailing the medical, economic, and social strains caused by them and the need for multidisciplinary pain management strategies to alleviate those strains has been observed. [18] Compared to the other types of pain analyzed in this study, chronic pain was the most researched among all journals. A recent study on the prevalence of chronic pain among adults in the US states that 50.2 million (20.5%) of the population reported pain on most days or every day. [19].

In the present study, we found that review articles were the most common type of study related to chronic pain. These literature reviews can be utilized to establish a base of knowledge, call attention to research gaps, and pave the path for future practices.

## Data Availability

No datasets were generated or analysed during the current study.
